# Association of *SMAD7* genetic markers and haplotypes with colorectal cancer risk

**DOI:** 10.1186/s12920-021-01150-3

**Published:** 2022-01-11

**Authors:** Maryam Alidoust, Leila Hamzehzadeh, Asma Khorshid Shamshiri, Fahimeh Afzaljavan, Mohammad Amin Kerachian, Azar Fanipakdel, Seyed Amir Aledavood, Abolghasem Allahyari, Alireza Bari, Hooman Moosanen Mozaffari, Ladan Goshayeshi, Alireza Pasdar

**Affiliations:** 1 Department of Medical Genetics and Molecular Medicine, Faculty of Medicine, Mashhad University of Medical Sciences, Mashhad, Iran; 2 Faculty of Medicine, Mashhad University of Medical Sciences, Mashhad, Iran; 3 Medical Genetics Research Center, Mashhad University of Medical Sciences, Mashhad, Iran; 4 Cancer Genetics Research Unit, Reza Radiotherapy and Oncology Center, Mashhad, Iran; 5 Cancer Research Center, Mashhad University of Medical Sciences, Mashhad, Iran; 6 Hematology and Oncology Department, Faculty of Medicine, Mashhad University of Medical Sciences, Mashhad, Iran; 7 Department of Gastroenterology and Hepatology, Faculty of Medicine, Mashhad University of Medical Sciences, Mashhad, Iran; 8 Surgical Oncology Research Center, Mashhad University of Medical Sciences, Mashhad, Iran; 9 Division of Applied Medicine, Medical School, University of Aberdeen, Foresterhill, Aberdeen, AB25 2ZD UK

**Keywords:** Colorectal cancer, GWAS, *SMAD7*, Polymorphisms, Association study

## Abstract

**Purpose:**

Colorectal cancer (CRC) is one of the common cancers with a high mortality rate worldwide. In Iran, there has been a trend of increased incidence of colorectal cancer in the last three decades that necessitates the early diagnosis. Genetic factors have an influential role in its etiology along with the conventional risk factors such as age, diet, and lifestyle. Results from GWAS have shown significant associations between *SMAD7* gene variants and risk of CRC. This study aimed to assess the association of certain polymorphisms as well as haplotypes of this gene and risk of colorectal cancer.

**Methods and materials:**

This study was designed as a case–control association study. After obtaining ethical approval and informed consent, blood samples from 209 patients with colorectal cancer were collected and DNA was extracted. Four variants: *rs4939827*, *rs34007497*, *rs8085824* and *rs8088297* were genotyped using ARMS-PCR method.

**Results:**

*SMAD7 rs4939827* in the recessive and co-dominant models was associated with colorectal cancer risk [TT/CT + CC: OR = 2.90, 95%CI (1.38–6.09), *p* = 0.005; CC + TT/CT: OR = 1.66, 95%CI (1.00–2.75), *p* = 0.01]. Haplotype analysis indicated that some SNP combinations including two for-SNPs haplotypes of T-T-C-C and T-C-C-A were significantly associated with CRC risk.

**Conclusion:**

Based on the identified association of *SMAD7* gene variations and haplotypes with colorectal cancer risk in our population, genetic variations in this gene region may have a role in CRC development. This data may shed light on the genetic predisposition of CRC which involves different pathways including TGF-β.

**Supplementary Information:**

The online version contains supplementary material available at 10.1186/s12920-021-01150-3.

## Introduction

Cancer is a heterogenic disease and is one of the most significant health problems in developed, industrialized, and developing countries [1]. Colorectal cancer is the most common malignancy among gastrointestinal malignancies, and it accounts for 13% of all digestive tract malignancies [2]. Based on epidemiologic reports, 60% of CRCs occur in developed countries. However, this figure is continuously decreasing. It is in contrast to developing countries, particularly, Asian countries, where the incidence has been increasing during the two recent decades [3]. This disease has the third and fourth rank among cancers in men and women in incidence and mortality respectively [4]. Over the past three decades, in Iran, colorectal cancer incidence has been increased and reported as the third and fourth most common cancer type in males and females, respectively [5].

Several risk loci in Genome Wide Association Studies (GWAS) studies have indicated that they are associated with CRC risk. Some of these are involved in TGF-β signaling pathway. In this pathway, one of the most common polymorphisms associated with CRC risk is located on *SMAD7* gene [6].

It has been reported that the transforming growth factor beta signaling pathway controls some processes such as tumor initiation, cell proliferation, invasion and metastasis. This pathway has been considered as both a tumor suppressor pathway and a promoter of tumor invasion and progression [6]. *SMADs* are known as receptors and intracellular signal transducers mediating TGF-β signaling pathway [7]. *SMAD7* is a kind of inhibitory *SMADs,* and it is able to binned TGF-β receptor type I, so it acts as a negative regulator of the TGF-β signaling pathway. Therefore the TGF-β signaling is remarkably decreased by the act of *SMAD7,* which lead to increasing cancer risk. [8, 9].

One of the main problems in CRC is the late diagnosis, therefore, there is an increasing need in molecular studies which lead to the recognition of biological markers for early diagnosis [10]. Due to multifactorial nature of colorectal cancer, identifying genetic and environmental factors involved in its pathogenesis is would help to enhance our understanding for a better management of the disease [11]. The molecular variations related to a particular disorder may result in a new view for a preventive strategy. The development of genome characterization could result in advances in personalized medicine. Single Nucleotide Polymorphisms (SNP) have been shown as appropriate markers for forecasting susceptibility to various diseases such as colorectal cancer and can be considered as a road to personalized medicine [12].

Several studies have reported that influence of risk alleles on CRC risk, owing to allele distributions or specific Linkage Disequilibrium (LD) structure and specific genetic and environmental backgrounds, in various population is different. Therefore, identification of these variations could be a valuable tool for risk prediction in our population [13, 14]. In the present study, we conducted a case–control study to investigate the potential association of *SMAD7* gene polymorphisms with the risk of colorectal cancer in an Iranian population. Moreover, we assessed the association of these markers with the clinical features and tumor characteristics.

## Methods and materials

### Study populations

The study was performed in accordance with Declaration of Helsinki and relevant guidelines by the institutional ethics committee. The study was approved by the ethics committee of Mashhad University of Medical Sciences under the ethical approval number of (IR.MUMS.SM.REC.1396.234). The inclusion criteria determined which are as follows: 1- Any patients diagnosed with sporadic CRC who had Age ≥ 40 years. With consideration of the following exclusion criteria. 1-Familial CRC /strong family history of colorectal cancer were excluded. 2- Known genetic susceptibility syndromes (e.g., Lynch Syndrome, FAP) were excluded. 3- Inability to provide informed consent. In this case–control study, a total of 409 Iranian individuals in a period of time between 2016 and 2018 were enrolled. The case group included 209 patients with sporadic CRC diagnosis confirmed with positive colonoscopy and pathology result, and the control group consisted of 200 healthy controls who had normal colonoscopy results. We collected a 5-ml peripheral venous blood sample from each subject (all healthy controls and patients) after getting written informed consent.

### SNP selection DNA isolation and genotyping

In our study, selection of polymorphisms was performed based on several criteria; including validation of the association in numerous GWAS studies which denotes a strong association with colorectal cancer risk in different populations. We also considered selecting SNPs that are located in the same region to be able to perform haplotype analysis to examine the overall effect of these polymorphisms. We also considered selecting markers with an acceptable MAF and heterozygosity to achieve the highest possible study power. Therefore, based on these criteria, we selected four polymorphisms to have a more comprehensive picture of a possible association of *SMAD7* gene with CRC. Three of them were previously reported to be associated with colorectal cancer and are located in the intronic region (*rs4939827*, *rs34007497*, *rs8085824*) and *rs8088297* is located in 3′UTR. Minor allele frequencies of all SNPs were > 5%, and average heterozygosity was more than 0.3. Information related to the SNPs was obtained from the National Center of Biotechnology Information (NCBI) SNP database (www.ncbi.nlm.nih.gov/ SNP, dbSNP BUILD 156).

Collection of peripheral blood was done from each participant in EDTA coated vials and kept at − 80 °C. Genomic DNA extracted by the standard salting-out method. The quality of extracted DNA confirmed using 1% agarose gel electrophoresis containing Green Viewer. The concentration and purity of DNA were also evaluated utilizing Epoch™ Microplate Spectrophotometer (BioTek Instruments Inc., Winooski, VT, USA). ARMS (The Amplification-Refractory Mutation System) method was applied as the main genotyping technique. Primers were designed using Primr1 and WASP primer online software (www.primer1.soton.ac.uk/primer1.html, www.bioinfo.biotec.or.th/WASP/). The specifications of primers and set up protocols have been presented in Table 1. PCR reactions were performed in 10 µl total volume, comprising 1 µl of DNA (200 ng), 5 µl of Taq DNA Polymerase 2 × Master Mix (Ampliqon), 0.3 µl (10 μM) of each primer for *rs34007497* and *rs8085824*. 1.3 µl (10 μM) forward prime, 1.6 µl (10 μM) reverse primer and 1 µl (10 μM) of each control primer for *rs4939827*. And also 1 µl (10 μM) of each primer for *rs8088297*.

**Table 1:** Information for *SMAD7* SNPs primers

SNP	Primer sequence	Primer type	Tm	PCR product size	
*rs4939827*	CTCATCCAAAAGAGGACAC	Forward inner (C Allele)	54	140 bp	270 bp
	GTTTTCTGATTAACTCGCAGT	Reverse outer			
	ATTCACAAGGACCCTTGCT	Forward outer	58	167 bp	
	AGGGAGCTCTGGGGTCATA	Reverse inner (T Allele)			
*rs34007497*	AGACTCCTGCCTGCTCCTCTC	Forward inner (C Allele)	64	203 bp	553 bp
	TCCCCAGAAGGTGTGATGTCCA	Reverse outer			
	AGTGAGTGGAGTATGGACCTGAGATCG	Forward outer	64	393 bp	
	GGGGAGCTGGAGGGGACAGCCAC	Reverse inner (G Allele)			
*rs8085824*	GTCCCCCTTCTCCCCTGCC	Forward inner (C Allele)	62		191bp
	CGTCCCCCTTCTCCCCTGCT	Forward inner (T Allele)			
	CCTTACCAGAATCACCCCG	Reverse outer			
*rs8088297*	TAATAGCAGAGCTTAACACACAAGA	Forward inner (A Allele)	62	136 bp	309 bp
	TCACAGTATTGCTCACCCAGT	Reverse outer			
	TGAGCTAAGAACAGTGTCGAAGTA	Forward outer	70	217 bp	
	TCAGCACTGCCTGCTCCTAG	Reverse inner (C Allele)			

### Statistical analysis

Deviations from Hardy–Weinberg equilibrium was assessed by using the Chi-square test. Also The chi-square test was done for evaluation of allele and genotype frequencies between cases and control groups. Odds Ratios (OR) and corresponding 95% confidence intervals (95% CI) were calculated to estimate the conferred risk by the risky alleles. Results were also adjusted for potential confounders such as age, gender and BMI in the logistic regression analysis. To compare age and BMI between two groups (patients and healthy people), the Student’s t-test was performed. Associations between mentioned SNPs and CRC risk was evaluated for three different genetic models (dominant, recessive and allelic model) analysis. In addition, haplotype analysis was performed using PHASE v.2.1.1 software. The LD has been calculated by 2LD software version 1.00. All the statistical analyses were conducted using IBM SPSS Statistics for Windows, Version 16.0 (IBM, USA). *p* < 0.05 was considered to indicate statistical significance.

## Results

### Baseline characteristics

A total of 209 colorectal cancer cases and 200 controls were included in this study. The frequency distributions of baseline characteristics and histological parameters are presented in Table 2. In the healthy control group, the number of males and females was equal. No significant differences was indicated in the distribution of sex (*p* = 0.36) and age (*p* = 0.19) between cases and controls. Additionally, we investigated the correlations between BMI and the results showed a significant difference between cases and controls (*p* < 0.001). Regarding tumor status, the proportion of which patients were graded as “well-differentiated” was 11.0%, while moderate and poorly differentiated were 42.5% and 31.1% respectively.

**Table 2:** Baseline characteristics of patients (cases) and healthy (control) groups and tumor features

Characteristic	Cases	Controls	*p*-value	OR (95%CI)
Gender				
Female	95(45.5%)	100(50%)	Ref	
Male	114(54.5%)	100(50%)	0.358	0.83 (0.56–1.23)
Age of diagnosis^a^ (year)	56.84 ± 13.6	57.35 ± 8.4	0.192	0.99 (0.97–1.01)
Smoking status				
Positive	19(9.0%)	29(14.5%)	Ref	
Negative	156(74.6%)	171(85.5%)	0.179	0.65 (0.35–1.21)
Family history				
Positive	35(16.7%)	40(20.0%)	Ref	
Negative	131(62.6%)	160(80.0%)	0.321	0.78 (0.48–1.27)
BMI (Kg/m^2^)^b^	25.5 ± 4.8	27.8 ± 4.9	** < 0.001**	0.90 (0.86–0.95)
Tumor location				
Rectum	75 (35.8)			
Colon	59 (28.2)			
Sigmoid	43 (20.5)			
Grade				
Poor	65 (31.1)			
Moderate	89 (42.5)			
Well	23 (11.0)			

All statistically significant *P*-values are shown in bold

^a^Mean ± SD

^b^Body Mass Index

### Association between polymorphisms and the colorectal cancer risk

Table 3 included summarized data of the genotypic and allelic frequencies of four variants in the both case and control groups. The control group genotype distribution followed the Hardy–Weinberg equilibrium. We found a significant association between the *rs4939827* SNP and increased CRC risk [*p* = 0.02, OR = 2.49, 95% CI (1.13–4.49)]. This SNP was associated with CRC risk in the recessive and co-dominant genetic models [TT/CT + CC: OR = 2.90, 95% CI (1.38–6.09), *p* = 0.005; CC + TT/CT: OR = 1.66, 95% CI (1.00–2.75), *p* = 0.01].

**Table 3:** Distribution of *SMAD7* genotypes and allele frequency among colorectal cancer patients and controls

Genetic model (HWE)	Genotype	Cases (n = 209)	Controls (n = 200)	*p*-value	OR (95%CI)	*p*-value_adj.*_	OR (95%CI) _adj.*_
***rs4939827*** (0.1)	CC	89(42.6%)	78(40.0%)			Ref	
	CT	83(39.7%)	101(51.8%)	0.12	0.72(0.47–1.09)	0.294	0.75(0.43–1.29)
	TT	37(17.7%)	16(8.2%)	**0.036**	2.02(1.04–3.92)	**0.024**	2.49(1.13–4.49)
Dominant model	TT + CT	120 (57.4%)	117 (60.0%)			Ref	
	CC	89(42.6%)	78(40.0%)	0.598	1.11(0.75–1.65)	0.920	1.03(0.62–1.70)
Recessive model	CC + CT	172 (82.3%)	179 (91.8%)			Ref	
	TT	37(17.7%)	16(8.2%)	**0.006**	2.40(1.29–4.48)	**0.005**	2.90(1.38–6.09)
Multiplicative model	C	157(37.6%)	133(34.1%)			Ref	
	T	261(62.4%)	257(65.9%)	0.350	0.87 (0.65–1.16)	0.189	0.78 (0.54–1.13)
Co-dominant model	CT	83(39.7%)	101(51.8%)			Ref	
	CC + TT	126 (60.3%)	94 (48.2%)	**0.015**	1.63 (1.10–2.42)	0.049	1.66 (1.00–2.75)
***rs34007497*** (0.98)	CC	135 (64.6%)	126 (63.0%)			Ref	
	CG	52 (24.9%)	66 (33.0%)	0.168	0.73(0.47–1.14)	0.183	0.68 (0.39–1.20)
	GG	22 (10.5%)	8 (4.0%)	**0.029**	2.56 (1.10–5.97)	0.327	1.71 (0.558–5.04)
Recessive model	CC + CG	187 (89.5%)	192 (96.0%)			Ref	
	GG	22 (10.5%)	8 (4.0%)	**0.015**	2.82(1.22–4.50)	0.227	1.93 (0.66–5.59)
Dominant model	GG + CG	74 (35.4%)	74 (37.0%)			Ref	
	CC	135 (64.6%)	126 (63.0%)	0.737	1.07(0.71–1.60)	0.379	0.79 (0.47–1.33)
Multiplicative model	C	96 (22.9%)	82 (20.5%)			Ref	
	G	322 (77.1%)	318 (79.5%)	0.393	1.16(0.83–1.63)	0.788	0.94 (0.61–1.46)
Co-dominant model	CC + GG	157 (75.1%)	134 (67.0%)			Ref	
	CG	52 (24.9%)	66 (33.0%)	0.071	0.67 (0.44–1.03)	0.135	0.65 (0.37–1.14)
***rs8085824*** (0.53)	TT	58 (28.9%)	77 (38.5%)			Ref	
	CT	97 (48.3%)	100 (50.0%)	**0.013**	0.48 (0.27–0.86)	0.092	0.53 (0.26–1.11)
	CC	46 (22.8%)	23 (11.5%)	**0.002**	2.06 (1.45–4.86)	0.086	1.95 (0.91–4.20)
Recessive model	CT + TT	155 (77.1%)	177 (88.5%)			Ref	
	CC	46 (22.9%)	23 (11.5%)	**0.042**	1.54 (1.02–2.34)	0.477	1.21 (0.71–2.07)
Dominant model	CC + CT	143 (71.1%)	123 (61.5%)			Ref	
	TT	58 (28.9%)	77 (38.5%)	**0.003**	0.44 (0.25–0.75)	0.067	0.52 (0.26–1.05)
Multiplicative model	T	189 (56.4%)	213 (45.6%)			Ref	
	C	146 (43.6%)	254 (54.4%)	**0.003**	0.65 (0.49–0.86)	0.150	0.76 (0.53–1.10)
Co-dominant model	CC + TT	104 (51.7%)	100 (50.0%)			Ref	
	CT	97 (48.3%)	100 (50.0%)	0.727	0.93 (0.63–1.38)	0.537	0.85 (0.51–1.42)
***rs8088297*** (0.67)	CC	3 (1.4%)	3 (1.5%)			Ref	
	AC	15 (7.2%)	33 (16.5%)	0.367	0.35 (0.08–2.52)	0.306	0.35 (0.05–2.62)
	AA	191 (91.4%)	164 (82.0%)	0.853	1.16 (0.23–5.85)	0.645	1.55 (0.23–10.33)
Recessive model	CC + AC	18 (8.6%)	36 (18.0%)			Ref	
	AA	191 (91.4%)	164 (82.0%)	**0.006**	2.33 (1.27–4.26)	0.203	1.62 (0.77–3.40)
Dominant model	AA + AC	206 (98.6%)	197 (98.5%)			Ref	
	CC	3 (1.4%)	3 (1.5%)	0.957	0.96 (0.19–4.79)	0.587	0.59 (0.09–3.92)
Multiplicative model	C	21 (5.02%)	39 (9.7%)			Ref	
	A	397 (94.98%)	361 (90.3%)	**0.011**	2.04 (1.18–3.54)	0.319	1.41 (0.72–2.75)
Co-dominant model	CC + AA	194 (92.8%)	167 (83.5%)			Ref	
	AC	15 (7.2%)	33 (16.5%)	**0.004**	0.39 (0.20–0.74)	0.129	0.54 (0.24–1.20)

All statistically significant *P*-values are shown in bold

^*^Adjusted for age, sex and BMI

**Fig. 1:**
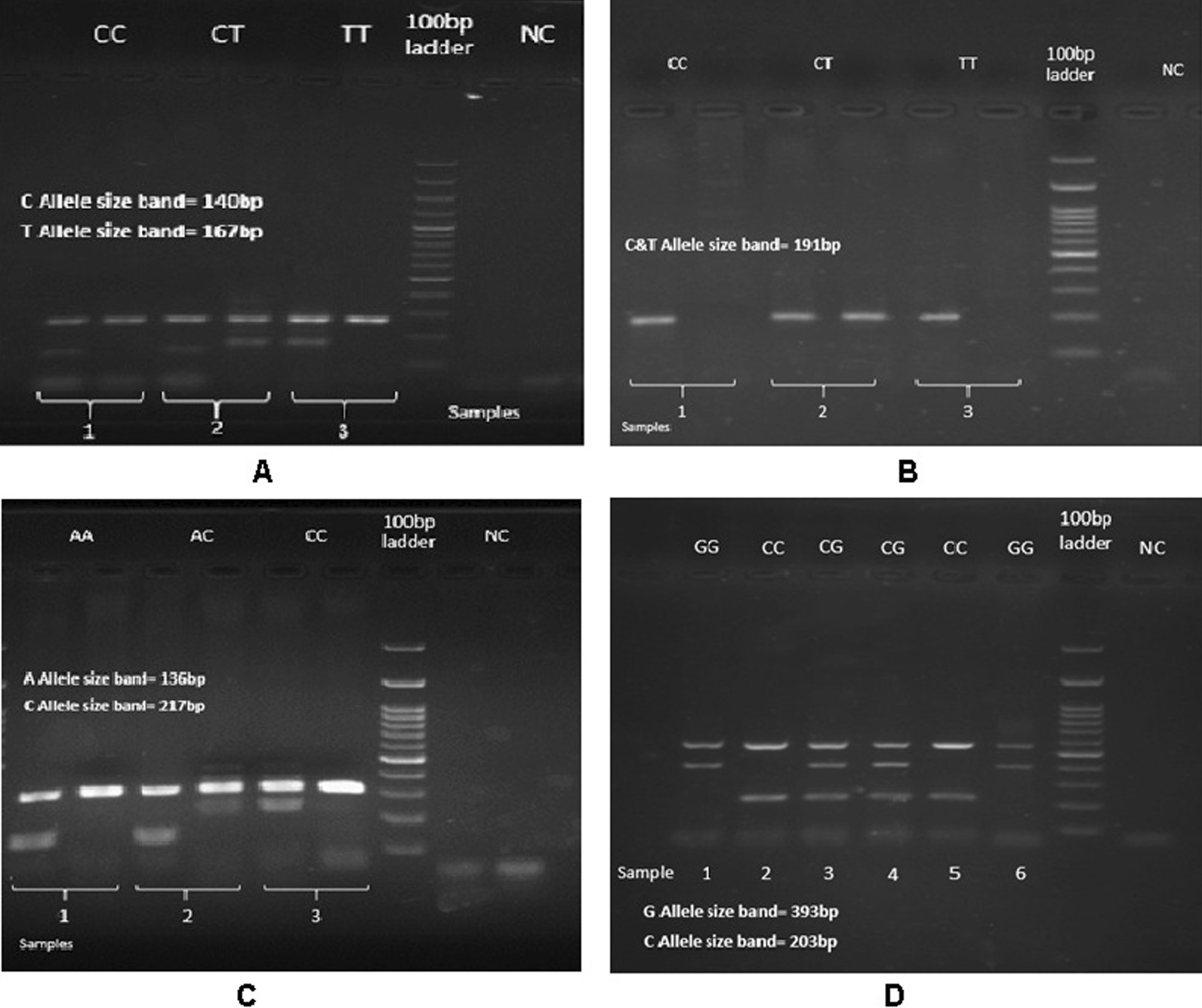
Agarose gel electrophoresis (2%) showing various genotypes of four polymorphisms as follows: **A,** Represent *rs4939827*, sample one; CC genotype (140 bp), sample 2; CT genotype (140, 167 bp) and Sample 3; TT genotype (167 bp). **B,** Represent* rs8085824*, sample one; CC genotype, sample 2; CT genotype and Sample 3; TT genotype(C & T Allel size band 191 bp). **C,** Represent *rs8088297*, sample one; AA genotype (136 bp), sample 2; AC genotype (136, 217 bp) and Sample 3; CC genotype (217 bp). **D,** Represent *rs34007497*, sample 1, 6; GG genotype (393 bp), sample 3, 4; CG genotype (203, 393 bp) and Sample 2, 5; CC genotype (203 bp)

The results showed a significant association between the other three polymorphisms and colorectal cancer.*rs34007497* was associated with the risk of disease in recessive genetic model (GG/CC + CG, *p* = 0.01). *rs8085824* indicated the association with colorectal cancer in dominant (CC + CT/TT, *p* = 0.003), recessive (CC/CT + TT, *p* = 0.04) and allelic (T/C, *p* = 0.003) genetic models. Furthermore, *rs8088297* was associated with colorectal cancer risk in recessive (CC + AC/AA: OR = 2.33, *p* = 0.006), co-dominant (AC/CC + AA, *p* = 0.004) and allelic (A/C, *p* = 0.01) genetic models. Although after adjustment for age, gender and BMI as a confounder factor, there was no association between these three polymorphisms and colorectal cancer risk. Electrophoresis results of polymorphisms genotyping are shown in Fig. 1.

Haplotype analysis showed two haplotypes, T-C haplotype of *rs8085824- rs34007497*, as well as T-C and C-T haplotypes of *rs8085824- rs4939827* were associated with CRC risk. In addition, C-C haplotype of *rs8085824- rs4939827* conferred a decreased risk of CRC*.* On the other hand, in haplotypes consisting of *rs8085824* - *rs34007497*- *rs4939827* the distribution of three haplotypes C-C-T, C-C-C and T-C-C were different between patients and healthy groups. Also, A-T-C and A-C-T haplotypes of *rs8088297* - *rs8085824*- *rs4939827* revealed an elevated risk of CRC. The results have been shown in details in Table 4. In four-SNP haplotype, three haplotypes including T-T-C-C, T-C-C-A and T-C-C-C were associated with risk of CRC. No significant association with the risk of CRC show in other haplotypes which are shown in (Additional file 1 - haplotypes distribution; Table S1).

**Table 4:** Distribution of associated haplotypes with risk of colorectal cancer

Polymorphisms	Haplotype	Case	Control	*p*-value _adj*_	OR (95%CI) _adj.*_
*rs8085824-rs34007497*	Others	289 (69.1%)	310 (77.5%)	Ref	
	T-C	129 (30.9%)	90 (22.5%)	**0.029**	1.55 (1.05–2.95)
*rs8085824-rs4939827*	Others	332 (79.4%)	362 (90.5%)	Ref	
	T-C	86 (20.6%)	38 (9.5%)	**0.005**	2.05 (1.24–3.42)
	Others	368 (88.0%)	373 (93.2%)	Ref	
	C-T	50 (12.0%)	27 (6.8%)	**0.001**	2.67 (1.51–4.72)
	Others	243 (58.1%)	173 (43.3%)	Ref	
	C-C	175 (41.9%)	227 (56.7%)	**0.002**	0.56 (0.40–0.80)
*rs8088297-rs8085824-rs34007497*	Others	292 (69.9%)	312 (78.0%)	Ref	
	A-T-C	126 (30.1%)	88 (22.0%)	**0.047**	1.49 (1.00–2.22)
*rs8088297-rs8085824-rs4939827*	Others	329 (78.7%)	364 (91.0%)	Ref	
	A-T-C	89 (21.3%)	36 (9.0%)	**0.001**	2.35 (1.42–3.89)
	Others	369 (88.3%)	373 (93.2%)	Ref	
	A-C-T	49 (11.7%)	27 (6.8%)	**0.002**	2.52 (1.42–4.46)
	Others	262 (62.7%)	198 (49.5%)	Ref	
	A-C-C	156 (37.3%)	202 (50.0%)	**0.002**	0.57 (0.40–0.82)
*rs8085824-rs34007497-rs4939827*	Others	357 (85.4%)	371 (92.7%)	Ref	
	T-C-C	61 (14.6%)	29 (7.3%)	**0.002**	2.38 (1.36–4.15)
	Others	374 (89.5%)	374 (93.5%)	Ref	
	C-C-T	44 (10.5%)	26 (6.5%)	**0.004**	2.40 (1.33–4.35)
	Others	269 (64.4%)	198 (49.5%)	Ref	
	C-C-C	149 (35.6%)	202 (50.5%)	**0.001**	0.54 (0.38–0.77)
*rs4939827-rs34007497-rs8085824-rs8088297*	Others	358 (85.6%)	373 (93.2%)	Ref	
	T-T-C-C	60 (14.4%)	27 (6.8%)	**0.002**	2.42 (1.37–4.27)
	Others	374 (89.5%)	375 (93.8%)	Ref	
	T-C-C-A	44 (10.5%)	25 (6.2%)	**0.001**	2.63 (1.45–4.75)
	Others	275 (65.8%)	221 (55.2%)	Ref	
	T-C-C-C	143 (34.2%)	179 (44.8%)	**0.009**	0.61 (0.43–0.88)

All statistically significant *P*-values are shown in bold

^*^Adjusted for age, sex and BMI

## Discussion

The role of TGF-β signaling has proven to be important in homeostasis, cell differentiation and tumor suppression [15, 16]. Additionally, it is a pleiotropic cytokine which has a dual function in cancer development: it acts as a tumor suppressor and a tumor promoter in the early stages and late stages [17]. The production of TGF-β increased in various tumor types, such as CRC [18]. Regulation of TGF-β by *SMAD7* is critical to preserve gastrointestinal homeostasis. *SMAD7* as an intracellular antagonist of TGF-β signaling binned to the receptor compound and act as an obstacle for initiation of downstream signaling proceedings [19, 20]. *SMAD7* interacts with activated TGF-β type I receptor, thereby blocking the phosphorylation and activation of *SMAD2* thus resulting in lack of formation of SMAD2/SMAD4 complexes and ultimately inhibiting binding to transcription factors and expression of target genes by preventing of entering the complex (SMAD2/SMAD4) into the nucleus [6]. The influence of *SMAD7* expression has been proven in CRC progression. High-level expression of *SMAD7* mRNA in CRC cell lines was reported to boost cell growth and prevent apoptosis via a mechanism dependent on the suppression of TGF-β signaling pathway [21]. Several investigations have examined numerous genetic variations within *SMAD7* gene (on 18q21). Broderick et al. performed a genome-wide association study which revealed that the *SMAD7* intron 3 was highly polymorphic, harboring one of the associated SNP- *rs4939827* [22]. However, most of the association studies have been performed on European Caucasian populations. Because of differences in the incidence of CRC and the allele distribution of SNPs across populations, this study investigated four candidate variants to examine the association of these SNPs and CRC risk in an Iranian population. We also evaluated the association of these polymorphisms with clinicopathological characteristics such as age, sex, tumor location, tumor grade and family history.

The three polymorphisms *rs4939827* (C > T), *rs34007497* (C > G) and *rs8085824* (C > T) are located in the intronic region of the *SMAD7* gene. Our results suggest a significant association in the genotype distribution of *rs4939827* between cases and controls. Previous studies have also mainly shown the association between this SNP and the risk of CRC [22–24]. Our study demonstrated that *rs4939827* TT genotype increases the risk of CRC. The results of our study were in line with a meta-analysis by Huang Y. et al. including 37 studies on *rs4939827* (48,751 cases and 61,529 controls) [25]. They found an association between this SNP and colorectal cancer risk with a 15% increase in the risk of disease. Although in our study, we did not find any significant association between the above mentioned polymorphisms and CRC susceptibility, even when stratifying the samples by age, gender, tumor site or family history. Mates and colleagues demonstrated that carriers of the T allele had an almost fivefold greater risk of developing rectal cancer compared with colon cancer [26]. Regarding the function of selected polymorphisms, none of four SNP in this study has a specific function.

At first, we found a statistically significant association between the *rs34007497*, *rs8085824* and *rs8088297* with the risk of CRC in our study.

However, after adjustment for some confounder factors (age, sex and BMI) in different genetic models the associations of these three polymorphisms did not remain significant. Therefore, we can conclude that these three markers were not associated independently with colorectal cancer risk.

*rs8088297* (A > C) is located in the 3′UTR region of the *SMAD7* gene. Since The 3′ and 5′-UTRs of mRNAs have a regulating role, so it is crucial. mRNA folding is affected by sequence alterations in the UTR regions. SNPs in 3′-UTRs can change target recognition of microRNAs by disrupting sequence complementarity [27]. Super-enhancers define a cell’s identity as large enhancer clusters which in several studies approximately whole of the human *SMAD7* gene categorized as a super-enhancer in colon crypts, sigmoid colon and fetal intestine [28].

Haplotype-based association analysis has numerous advantages over one-SNP approach. First, the loss of information, imputable to bi-allelic rather than multi-allelic loci, can be overcome by assessing haplotypes rather than single-locus tests. Second, methods based on haplotypes are regarded as more powerful than those based on single markers. Third, haplotype-based methods can potentially detect associations of *cis*-interactions SNPs that are among nearby SNPs. In light of these aforementioned benefits, we performed a haplotype analysis [29]. In our study we found a significant association of several haplotype combinations [(*rs8085824*-*rs34007497*- *rs4939827*); (*rs8088297*-*rs8085824*-*rs4939827*); (*rs8088297*-*rs8085824*-*rs34007497*); (*rs8085824- rs34007497);* (*rs8085824*-*rs4939827*); (*rs4939827*-*rs34007497*-*rs8085824*-*rs8088297*)] with CRC risk. It can reflect the effect of multiple SNPs on the risk of the disease which remained significant even after adjustment for confounders (age, sex, BMI). Therefore, the haplotype analysis further illustrated the pivotal role of these variations in combination with each other where they can confer some degrees of risk.

As mentioned before, we selected a variety of polymorphisms on *SMAD*7 genes in different positions to have an overview of the whole gene. One of the strengths of this study is the evaluation of several markers across the gene and haplotype analysis which can increase the study power. To the best of our knowledge, none of the previous studies looked at haplotypes of this gene in terms of association with CRC. Moreover, replication of association of the *rs4939827* With CRC in our population can strengthen the evidence of the effect of this genetic risk factor on CRC which can be used in clinical risk assessment, especially in direct-to-consumer genetic testing.

The development of genome characterization could result in advances in personalized medicine. Variations have been shown as appropriate markers for forecasting susceptibility to various diseases such as colorectal cancer and can be considered as a road to personalized medicine.

## Conclusion

To the best of our knowledge, this is the first case–control study which has assessed the impact of these four SNPs on clinicopathological features and CRC risk in an Iranian population. Our results suggest that the *rs4939827* is associated with CRC susceptibility in the Iranian population. Future large population-based studies are needed to confirm the accuracy of findings and to explore gene–environment interactions as well. Ultimately, future studies looking at the functional activity of these variations and their influence on tumorigenesis would help us to realize the principal mechanisms involved in CRC development.

## Supplementary Information

**Additional file 1.** Table S1. Distribution of *SMAD7* Haplotypes among Colorectal Cancer Patients and Controls.

## Data Availability

The datasets created during the current study are not publicly accessible due to the possibility of compromising the privacy of individuals. According to the written approval forms accepted by the Ethics Committee of the Mashhad University of Medical Sciences (MUMS), the data will only be available to researchers within project. The data would be available upon request from the corresponding authors (according to the MUMS rules and regulations).
