# Supplementary material for: Association of SMAD7 genetic markers and haplotypes with colorectal cancer risk
Source: BMC Med Genomics. 2022 Jan 11;15:8. doi: 10.1186/s12920-021-01150-3 (PMC8753827; doi:10.1186/s12920-021-01150-3)
Supplement: Supplementary file 1 — Additional file 1. Table S1. Distribution of SMAD7 Haplotypes among Colorectal Cancer Patients and Controls. [file 12920_2021_1150_MOESM1_ESM.docx]

**Table S1. Distribution of *SMAD7* Haplotypes among Colorectal Cancer Patients and Controls**

| **Polymorphism’s combination** | **Haplotypes** | **Case** | **Control** | **P-value _Adj_.*** | **OR (95%CI) _Adj_*** |
| --- | --- | --- | --- | --- | --- |
| rs34007497-rs4939827 | Others | 370 (88.5%) | 351 (87.8%) | Ref. |  |
|  | G-T | 48 (11.5%) | 49 (12.3%) | 0.733 | 0.90 (0.53-1.57) |
|  | Others | 370 (88.5%) | 367 (91.8%) | Ref. |  |
|  | G-C | 48 (11.5%) | 33 (8.3%) | 0.994 | 1.00 (0.54-1.87) |
|  | Others | 309 (73.9%) | 312 (78.0%) | Ref. |  |
|  | C-T | 109 (26.1%) | 88 (22.0%) | 0.088 | 1.42 (0.95-2.12) |
|  | Others | 205 (49.0) | 170 (42.5%) | Ref. |  |
|  | C-C | 213 (51.0%) | 230 (57.5%) | 0.212 | 0.80 (0.56-1.14) |
|  |  |  |  |  |  |
| rs8085824-rs34007497 | Others | 350 (83.7%) | 344 (86.0%) | Ref |  |
|  | T-G | 68 (16.3%) | 56 (14.0%) | 0.623 | 1.14 (0.68-1.91) |
|  | Others | 289 (69.1%) | 310 (77.5%) | Ref |  |
|  | T-C | 129 (30.9%) | 90 (22.5%) | **0.029** | 1.55 (1.05-2.95) |
|  | Others | 390 (93.3%) | 374 (93.5%) | Ref |  |
|  | C-G | 28 (6.7%) | 26 (6.5%) | 0.810 | 1.09 (0.55-2.16) |
|  | Others | 225 (53.8%) | 172 (43.0%) | Ref |  |
|  | C-C | 193 (46.2%) | 228 (57.0%) | 0.089 | 0.74 (0.52-1.05) |
|  |  |  |  |  |  |
| rs8085824-rs4939827 | Others | 311 (74.4%) | 292 (73.0%) | Ref |  |
|  | T-T | 107 (25.6%) | 108 (27.0%) | 0.415 | 0.84 (0.56-1.27) |
|  | Others | 332 (79.4%) | 362 (90.5%) | Ref |  |
|  | T-C | 86 (20.6%) | 38 (9.5%) | **0.005** | 2.05 (1.24-3.42) |
|  | Others | 368 (88.0%) | 373 (93.2%) | Ref |  |
|  | C-T | 50 (12.0%) | 27 (6.8%) | **0.001** | 2.67 (1.51-4.72) |
|  | Others | 243 (58.1%) | 173 (43.3%) | Ref |  |
|  | C-C | 175 (41.9%) | 227 (56.7%) | **0.002** | 0.56 (0.40-0.80) |
|  |  |  |  |  |  |
| rs8088297-rs8085824 | Others | 227 (54.3%) | 261 (65.2%) | Ref |  |
|  | A-T | 191 (45.7%) | 139 (34.8%) | 0.140 | 1.31 (0.91-1.88) |
|  | Others | 212 (50.7%) | 178 (44.5%) | Ref |  |
|  | A-C | 206 (49.3%) | 222 (55.5%) | 0.380 | 0.85 (0.60-1.21) |
|  | Others | 402 (96.2%) | 368 (92.0%) | Ref |  |
|  | C-C | 16 (3.8%) | 32 (8.0%) | 0.275 | 0.66 (0.31-1.39) |
|  |  |  |  |  |  |
| rs8088297-rs34007497 | Others | 331 (79.2%) | 332 (83.0%) | Ref |  |
|  | A-G | 87 (20.8%) | 68 (17.0%) | 0.978 | 1.01 (0.63-1.60) |
|  | Others | 108 (25.8%) | 107 (26.8%) | Ref |  |
|  | A-C | 310 (74.2%) | 293 (73.2%) | 0.542 | 1.13 (0.76-1.70) |
|  | Others | 406 (97.1%) | 375 (93.7%) | Ref |  |
|  | C-C | 12 (2.9%) | 25 (6.3%) | 0.476 | 0.74 (0.32-1.70) |
|  |  |  |  |  |  |
| rs8088297-rs4939827 | Others | 265 (63.4%) | 268 (67.0%) | Ref |  |
|  | T-T | 153 (36.6%) | 132 (33.0%) | 0.231 | 1.25 (0.87-1.80) |
|  | Others | 174 (41.6%) | 171 (42.8%) | Ref |  |
|  | T-C | 244 (58.4%) | 229 (57.2%) | 0.553 | 1.13 (0.78-1.58) |
|  | Others | 401(95.9%) | 367 (91.7%) | Ref |  |
|  | C-C | 17 (4.1%) | 33 (8.3%) | 0.247 | 0.64 (0.30-1.36) |
|  |  |  |  |  |  |
| rs8088297-rs8085824-rs34007497 | Others | 354 (84.7%) | 352 (88.0%) | Ref |  |
|  | A-T-G | 64 (15.3%) | 48 (12.0%) | 0.754 | 1.09 (0.63-1.89) |
|  | Others | 292 (69.9%) | 312 (78.0%) | Ref |  |
|  | A-T-C | 126 (30.1%) | 88 (22.0%) | **0.047** | 1.49 (1.00-2.22) |
|  | Others | 395 (94.5%) | 378 (94.5%) | Ref |  |
|  | A-C-G | 23 (5.5%) | 22 (5.5%) | 0.772 | 1.12 (0.53-3.34) |
|  | Others | 234 (56.0%) | 197 (49.3%) | Ref |  |
|  | A-C-C | 184 (44.0%) | 203 (50.2%) | 0.271 | 0.82 (0.58-1.17) |
|  |  |  |  |  |  |
| rs8088297-rs8085824-rs4939827 | Others | 315 (75.4%) | 300 (75.0%) | Ref |  |
|  | A-T-T | 103 (24.6%) | 100 (25.0%) | 0.540 | 0.88 (0.58-1.33) |
|  | Others | 329 (78.7%) | 364 (91.0%) | Ref |  |
|  | A-T-C | 89 (21.3%) | 36 (9.0%) | **0.001** | 2.35 (1.42-3.89) |
|  | Others | 369 (88.3%) | 373 (93.2%) | Ref |  |
|  | A-C-T | 49 (11.7%) | 27 (6.8%) | **0.002** | 2.52 (1.42-4.46) |
|  | Others | 262 (62.7%) | 198 (49.5%) | Ref |  |
|  | A-C-C | 156 (37.3%) | 202 (50.0%) | **0.002** | 0.57 (0.40-0.82) |
|  | Others | 403 (96.4%) | 371 (92.7%) | Ref |  |
|  | C-C-C | 15 (3.6%) | 29 (7.3%) | 0.254 | 0.63 (0.29-1.39) |
|  |  |  |  |  |  |
| rs8088297-rs34007497-rs4939827 | Others | 376 (90.0%) | 361 (90.2%) | Ref |  |
|  | A-G-T | 42 (10.0%) | 39 (9.8%) | 0.805 | 1.08 (0.59-1.96) |
|  | Others | 373 (89.2%) | 371 (92.7%) | Ref |  |
|  | A-G-C | 45 (10.8%) | 29 (7.3%) | 0.754 | 1.11 (0.58-2.13) |
|  | Others | 310 (74.2%) | 314 (78.5%) | Ref |  |
|  | A-C-T | 108 (25.8%) | 86 (21.5%) | 0.103 | 1.40 (0.93-2.10) |
|  | Others | 216 (51.7%) | 193 (48.2%) | Ref |  |
|  | A-C-C | 202 (48.3%) | 207 (51.8%) | 0.393 | 0.86 (0.60-1.22) |
|  |  |  |  |  |  |
| rs8085824-rs34007497-rs4939827 | Others | 379 (90.7%) | 353 (88.2%) | Ref |  |
|  | T-G-T | 39 (9.3%) | 47 (11.8%) | 0.218 | 0.68 (0.37-1.25) |
|  | Others | 389 (93.1%) | 391 (97.8%) | Ref |  |
|  | T-G-C | 29 (6.9%) | 9 (2.3%) | 0.235 | 1.80 (0.68-4.74) |
|  | Others | 350 (83.7%) | 339 (84.8%) | Ref |  |
|  | T-C-T | 68 (16.3%) | 61 (15.2%) | 0.953 | 1.01 (0.63-1.64) |
|  | Others | 357 (85.4%) | 371 (92.7%) | Ref |  |
|  | T-C-C | 61 (14.6%) | 29 (7.3%) | **0.002** | 2.38 (1.36-4.15) |
|  | Others | 396 (94.7%) | 375 (93.8%) | Ref |  |
|  | C-G-C | 22 (5.3%) | 25 (6.2%) | 0.586 | 0.81 (0.37-1.74) |
|  | Others | 374 (89.5%) | 374 (93.5%) | Ref |  |
|  | C-C-T | 44 (10.5%) | 26 (6.5%) | **0.004** | 2.40 (1.33-4.35) |
|  | Others | 269 (64.4%) | 198 (49.5%) | Ref |  |
|  | C-C-C | 149 (35.6%) | 202 (50.5%) | **0.001** | 0.54 (0.38-0.77) |
|  |  |  |  |  |  |
| rs4939827-rs34007497-rs8085824-rs8088297 | Others | 384 (91.9%) | 364 (91.0%) | Ref |  |
|  | T-T-G-A | 34 (8.1%) | 36 (9.0%) | 0.254 | 0.67 (0.34-1.33) |
|  | Others | 389 (93.1%) | 390 (97.5%) | Ref |  |
|  | T-T-G-C | 29 (6.9%) | 10 (2.5%) | 0.320 | 1.61 (0.63-4.14) |
|  | Others | 355 (84.9%) | 339 (84.7%) | Ref |  |
|  | T-T-C-A | 63 (15.1%) | 61 (15.3%) | 0.522 | 0.85 (0.51-1.40) |
|  | Others | 358 (85.6%) | 373 (93.2%) | Ref |  |
|  | T-T-C-C | 60 (14.4%) | 27 (6.8%) | **0.002** | 2.42 (1.37-4.27) |
|  | Others | 403 (96.4%) | 380 (95.0%) | Ref |  |
|  | T-C-G-C | 15 (3.6%) | 20 (5.0%) | 0.502 | 0.74 (0.30-1.80) |
|  | Others | 374 (89.5%) | 375 (93.8%) | Ref |  |
|  | T-C-C-A | 44 (10.5%) | 25 (6.2%) | **0.001** | 2.63 (1.45-4.75) |
|  | Others | 275 (65.8%) | 221 (55.2%) | Ref |  |
|  | T-C-C-C | 143 (34.2%) | 179 (44.8%) | **0.009** | 0.61 (0.43-0.88) |

*Adjusted for age, sex and BMI

All statistically significant *p*-values are shown in bold
